# Reproductive-Stage Heat Stress in Cereals: Impact, Plant Responses and Strategies for Tolerance Improvement

**DOI:** 10.3390/ijms23136929

**Published:** 2022-06-22

**Authors:** Tinashe Zenda, Nan Wang, Anyi Dong, Yuzhi Zhou, Huijun Duan

**Affiliations:** 1 State Key Laboratory of North China Crop Improvement and Regulation, Hebei Agricultural University, Baoding 071001, China; tzenda@hebau.edu.cn (T.Z.); wangnan@hebau.edu.cn (N.W.); donganyi@hebau.edu.cn (A.D.); 2 Department of Crop Genetics and Breeding, College o Agronomy, Hebei Agricultural University, Baoding 071001, China; 3 Library Department, Hebei Agricultural University, Baoding 071001, China; yzzhou1967@sina.com

**Keywords:** heat stress (HS), cereal crops, reproductive stage, HS response mechanisms, phytohormonal regulation, epigenetic regulation, HS improvement strategies

## Abstract

Reproductive-stage heat stress (RSHS) poses a major constraint to cereal crop production by damaging main plant reproductive structures and hampering reproductive processes, including pollen and stigma viability, pollination, fertilization, grain setting and grain filling. Despite this well-recognized fact, research on crop heat stress (HS) is relatively recent compared to other abiotic stresses, such as drought and salinity, and in particular, RSHS studies in cereals are considerably few in comparison with seedling-stage and vegetative-stage-centered studies. Meanwhile, climate change-exacerbated HS, independently or synergistically with drought, will have huge implications on crop performance and future global food security. Fortunately, due to their sedentary nature, crop plants have evolved complex and diverse transient and long-term mechanisms to perceive, transduce, respond and adapt to HS at the molecular, cell, physiological and whole plant levels. Therefore, uncovering the molecular and physiological mechanisms governing plant response and tolerance to RSHS facilitates the designing of effective strategies to improve HS tolerance in cereal crops. In this review, we update our understanding of several aspects of RSHS in cereals, particularly impacts on physiological processes and yield; HS signal perception and transduction; and transcriptional regulation by heat shock factors and heat stress-responsive genes. We also discuss the epigenetic, post-translational modification and HS memory mechanisms modulating plant HS tolerance. Moreover, we offer a critical set of strategies (encompassing genomics and plant breeding, transgenesis, omics and agronomy) that could accelerate the development of RSHS-resilient cereal crop cultivars. We underline that a judicious combination of all of these strategies offers the best foot forward in RSHS tolerance improvement in cereals. Further, we highlight critical shortcomings to RSHS tolerance investigations in cereals and propositions for their circumvention, as well as some knowledge gaps, which should guide future research priorities. Overall, our review furthers our understanding of HS tolerance in plants and supports the rational designing of RSHS-tolerant cereal crop cultivars for the warming climate.

## 1. Introduction

Plant heat stress (HS) refers to stress triggered by transient periods of elevated temperatures far exceeding optimal growth conditions that result in irreversible damages to cellular components, plant functioning and development through phenomena such as plasma membrane (PM) injury and liquidization, reactive oxygen species (ROS) overproduction and protein denaturation [1,2,3]. These phenomena lead to increased lipid peroxidation, compromised redox homeostasis, elevated metabolic imbalances and accelerated cell death, consequently inhibiting plant growth and development [4,5]. A new report, *Climate Change 2021: The Physical Science Basis*, reveals that global mean surface temperature continues to increase, with a high likelihood (>50%) of the global warming of 1.5 °C being reached or surpassed between 2021 and 2040 [6]. Consequently, **heat waves** (see Box 1) will occur with greater frequency and duration over most terrestrial regions [6]. This will have drastic repercussions on crop growth and productivity in cereal grain crops, such as maize (*Zea mays* L.), wheat (*Triticum aestivum* L.), rice (*Oryza sativa* L.), barley (*Hordeum vulgare* L.) and sorghum (*Sorghum bicolor* L.) [7], consequently impacting global food security [8,9].

In particular, the **reproductive stage** (R stage) HS (RSHS) poses a major constraint to cereal crop production globally by affecting **anthesis**, pollen viability and grain setting, photosynthesis, grain filling rate and duration, and ultimately, crop yields [2,10,11,12]. Frighteningly, under field conditions, HS seldom occur independently but co-exists with other abiotic stresses, especially drought, with the accumulated impact of their co-occurrence at the R stage being more detrimental than individual occurrences [13]. However, research efforts on HS tolerance are relatively recent as compared to studies on other abiotic stresses such as drought and salinity [14]. Additionally, most studies on HS tolerance in cereals have largely focused on the seedling and vegetative stages, while the R stage is the most sensitive to HS [11,12,15] and needs prioritization. Therefore, a better understanding of the molecular and physiological mechanisms underpinning RSHS response is a critical step toward developing effective strategies for HS tolerance improvement in cereals [16].

In coping with HS, plants have developed a suite of intricate and diverse short-term **acclimation** and long-term **adaptation** mechanisms at various levels, encompassing morphological, whole-plant, cellular, physiological, metabolic and molecular responses [4,17]. Chief among these strategies include altered cellular metabolism, phytohormonal regulation [18], and activation of stress-inducible genes, particularly antioxidant enzymes and Heat Shock Proteins (HSPs) [19]. Additionally, the roles of epigenetics, small RNAs (sRNAs), and post-translational modifications (PTMs) in thermotolerance have been recognized [5,20,21]. Therefore, uncovering various molecular and physiological mechanisms through which plants perceive, respond and adapt to HS offers crucial insights into designing effective strategies to improve RSHS tolerance in cereal crops [12,20].

The present review, therefore, updates our understanding on several aspects of RSHS in cereals, particularly its impact on physiological processes and yield; plant HS signal perception and transduction; and transcriptional regulation by heat shock factors and heat stress-responsive genes. We also discuss the regulation of plant HS tolerance by epigenetic mechanisms, post-translational modifications and **HS memory**. Additionally, we offer a critical set of strategies (encompassing genomics and plant breeding, transgenesis, **omics**, and agronomy) that could accelerate the development of RSHS resilient cereal crop cultivars. Furthermore, we highlight critical shortcomings to RSHS tolerance investigations in cereal crops and offer propositions for their circumvention, as well as some knowledge gaps, which should guide future research directions. Overall, our review furthers our understanding of RSHS tolerance in cereals and provides perspectives that support the rational designing of HS-tolerant cereal crops for the warming climate.

## 2. Impact of Reproductive-Stage Heat Stress (HS) in Cereals

The effect of HS depends upon the plant species or genotype, stage of crop development, extent of the stress and length of HS exposure [18]; for crop species-specific temperature thresholds, please see refs. [1,22,23,24,25,26]. In particular, the exposure of plants to above-optimum temperatures (usually by 10–15 °C [1]) at the R stage cause cytological alterations, including loss of membrane integrity, ROS accumulation, and altered carbohydrate and lipid metabolism in mature pollen, resulting in loss of pollen viability [17,19]. Additionally, leaf photosynthesis is severely affected, consequently impacting plant growth and development [27]. HS-induced damages to the photosystem II (PSII) within the thylakoid membranes of the chloroplasts decrease photosynthetic reactions, including electron transfer [1]. HS escalates ROS accumulation and oxidative stress; this leads to lipid peroxidation, increased organelles damage, and programmed cell death [4]. For example, HS (32/22 °C, day/night) during anthesis and grain filling in wheat decreased the photosynthetic rate (by 17 and 25%, respectively), grain yield per plant (by 29 and 44%, respectively), and increased thylakoid membrane damage (by 61 and 68%, respectively) compared to their respective control conditions (optimum temperatures, 22/14 °C) [28]. Moreover, HS during anthesis reduces fertility and disrupts photosynthesis mainly via accelerated leaf senescence, consequently reducing final grain yield (in terms of kernel number and kernel weight). Further, HS at anthesis and grain filling diminishes wheat grain quality [27].

In spring barley, ambient temperature shift from 20 °C/16 °C (day/night) to 28 °C/24 °C deferred inflorescence and spikelet development, and decreased floret number and grain per spike [29]. In rice, HS at the early R-stage impairs panicle initiation and spikelet development, leading to deformed floral organs, and reduced spikelet number and size, while HS at the later reproductive developmental stages, such as gametogenesis and anthesis, results in spikelet sterility and decreased yields [30]. Severe HS impacts meiosis, ovaries growth during pre-anthesis, synthesis and transfer of pollens at anthesis, consequently reducing the kernel number [2,10]. Additionally, HS during floral meristem development diminishes the overall sink size [31].

During the anthesis period, the RSHS-induced cytological alterations inflict drastic effects on anther dehiscence, pollen perception, pollen germination and development, pollen tube growth, fertilization and embryo formation processes [12]. For instance, under benign conditions, anther dehiscence entails anther wall opening through septum and stomium (special anther tissues) degeneration, precipitating the release of mature pollen grains from the anther locules, thereby effecting pollination [16]. However, HS causes anther indehiscence, for instance, in rice, thus interrupting pollen release [12]. Especially, anther and pollen development at anthesis are very sensitive to temperature instabilities, resulting in compromised reproduction and fertilization processes and, ultimately, reduced seed set and grain yields; structural abnormalities of pollen and pistil, and their decreased functionality, underline seed number reduction under HS [10,17,26]. Pre-anthesis HS (40–45 °C) significantly decreased the seed setting rate and grain quality in rice [32]. In wheat, HS (32/22 °C) during anthesis significantly decreased seed set (by 28%), number of grains spike^−1^ (by 36%) and grain yield plant^−1^ (by 29%) compared to optimum temperature (22/14 °C) [28]. In grain sorghum, HS (≥36/26 °C, day/night) caused decreased pollen production and viability, abortion of florets, and a remarkable decrease in seed size, which all contributed to the decreased grain yield [15]. Notably, pre-anthesis HS (>25 °C) decreased floret fertility, while HS (25–37 °C) at grain filling reduced individual grain weight (GW) and GW panicle^−1^ [15].

In addition, HS prolongs the anthesis-silking interval (ASI), negatively impacting pollen shedding. For instance, in maize, HS at pre-anthesis (40/30 ℃) and anthesis (36/26 ℃) advanced tasselling, shortened pollen shedding period, extended the ASI, and decreased the number and viability of pollen shed [33]. The shortened pollen shedding duration was as a result of the accelerated flowering speed of the tassel inflorescence [33]. Furthermore, post-anthesis HS hastens leaf senescence rate, and reduces the grain filling duration, resulting in smaller seed sizes and reduced yields in cereals [10,26]. In addition, HS may cause disruption of source-sink interactions, consequently impacting dry matter partitioning [26]. In terms of yield, a 1 °C rise in global mean ambient temperature is predicted to result in average global yield reductions of 6.0%, 3.2% and 7.4% in wheat, rice and maize, respectively [34]. However, these HS-induced crop productivity changes will vary with regions across the globe.

Box 1 **Key definitions.**

 **Acclimation:** The physiological alterations the plant makes to minimise the effects of the stress [35]. These strategies are typically displayed upon exposure to mildly elevated ambient temperature conditions (~27–29 °C for Arabidopsis) and are associated with growth and physiological development that facilitate optimal performance under suboptimal conditions.
 **Adaptation:** The process involving genetic and genome alterations within a population, which allows the population to accommodate a ‘new’ environment [35].
 **Anthesis:** The period when a flower is completely open and functional.
 **Heat escape:** A strategy involving rapid plant development, helping plants to cope with HS by completing their reproductive phase rapidly and maturing early, prior to the onset of harmful high temperatures. It often comes with small crop yield penalties [1].
 **Heat shock:** A sudden increase in temperature, resulting in drastic effects to plant functioning.
 **Heat warming:** A mild increase in temperature above the optimum level, which may or may not cause drastic effects to plant growth and functioning, depending with the species or genotype. It may have positive effects on plant growth and development.
 **Heatwave:** Generally, from a meteorological viewpoint, it describes a period of three or more consecutive days where the maximum temperature (*T_max_*) is over the 90th percentile for a particular location at a particular time [36].
 **Heat tolerance:** A special trait ensuring the maintenance of essential plant functions at high ambient temperatures (~30 °C and above for Arabidopsis) and contributes to the fitness and productivity of a genotype.
 **Haplotype:** A set of alleles (genomic region) within an organism co-inherited (as a block of variation) from a single parent.
 **Omics:** An array of tools applied to the study of large-scale data of a particular class or type of biological molecule in a cell, tissue, organ or whole organism. Inquiry can be conducted at different levels of molecular organization such as the genome, transcriptome, proteome, metabolome, phenome or their integration.
 **Reproductive stage:**  The period that commences with the transition of the vegetative meristem into an inflorescence and flower primordial and terminates as the seed reaches physiological maturity. It is a sequential process comprising several sub-stages, including floral induction, differentiation of inflorescence or flower parts, female and male meiosis, anthesis, pollination, fertilization and grain development to maturation [37].
 **Stress memory:** The ability of plants experiencing recurrent stress to activate the ‘stress imprint’ of the previous exposure and formulate an appropriate response to better deal with the recurring stressful conditions [38]. In other words, it implies a positive response to a second heat event.
 **Stress recovery:** The process of establishing new homeostasis after a period of HS exposure. The post-HS homeostasis may be reset to the pre-stress state or to a distinctly new state due to priming or HS memory formation [39].
 **Thermomophogenesis:**  A set of morphological and developmental alterations instituted to assist plants in withstanding suboptimal temperatures. It facilitates an optimal performance in suboptimal temperature conditions by enhancing cooling capacity [21].

## 3. Plant HS Response Mechanisms

To safeguard their survival and reproduction under adverse conditions, plants have evolved sophisticated mechanisms to sense and respond to environmental stress factors such as HS [40]. Here, these key HS response mechanisms are discussed.

### 3.1. Heat Stress Perception and Signal Transduction Cascades

#### 3.1.1. Plasma Membrane Embedded-, Ca^2+^- and ROS-Dependent Thermosensors and Signalling Pathways

For plants to develop HS adaptive strategies, first they need to sense it [40], with the PM being the primary site for stress-sensing [41]. PM-localized macromolecular sensors, viz., G-protein-coupled receptors (GPCRs), receptor-like kinases, microdomain NADPH-oxidases (also known as respiratory burst oxidase homologues, RBOHs), Ca^2+^ channels, and phospholipids perform this function [42]. The GPCRs perceive different secondary signals relayed from different stressors, including HS. This is mediated by GPCR binding to other ligands to yield conformational changes that activate heterotrimeric guanine nucleotide-binding proteins (G-proteins) [43]. The GPCRs–G proteins association stimulates small Ras-related GTP-binding proteins, which in turn initiate Ca^2+^ inositol triphosphate-mediated signalling pathway [44].

Additionally, the HS-exposed PM excites the activation of Ca^2+^ channels within it, leading to cytosolic Ca^2+^ levels modulation [3,40]. This cytosolic Ca^2+^ influx is prompted by HS-induced PM fluidization and regulated by a specific-type Ca^2+^ channel known as the Cyclic Nucleotide-Gated Channels (CNGCs) [19,45]. Moreover, cytosolic cyclic adenosine monophosphate (cAMP) levels are elevated. These cytosolic Ca^2+^ and cAMP influxes then trigger the induction of multiple downstream HS-signalling cascades, such as the Ca^2+^-dependent protein kinases (CDPKs), leading to the expression of transcriptional regulators [20,45].

ROS also play a critical role in HS signal transduction [3]. RBOHs orchestrate an apoplastic ROS (H_2_O_2_) surge under different stresses [30,46]. RBOH activity is regulated by intracellular Ca^2+^ build-up and is activated by Ca^2+^ via CDPKs or CBL-interacting protein kinase 26 (CIPK26)-mediated phosphorylation [42]. A surge of H_2_O_2_ within minutes of plant HS [46] suggests their role in HS response is closely tied to the sensor [40]. In HS-affected stomatal cells, apoplastic H_2_O_2_ enters the cytosol through aquaporins, such as the PM intrinsic proteins (PIPs) [44]. Similar to Ca^2+^, the PM H_2_O_2_ signals trigger retrograde (organelle to nucleus) signalling and HS gene expression [42].

Nitric oxide (NO) also transiently accumulates in plant response to HS [47]. Although mechanisms of NO regulation of plant HS response are still abstract [18], accruing evidence suggests that NO mediates NO-ROS crosstalk that regulates HS signalling and protein PTMs [3,18] and functions downstream of H_2_O_2_ signalling [48] and upstream of CaM3 [47] in basal thermotolerance regulation. These findings can imply that a feedback loop that modulates NO, ROS and Ca^2+^ signalling exists, as part of a complex gene regulatory network (GRN) that respond to HS, probably through induction of HSF binding to target downstream HS-responsive genes [3,49]. In addition to Ca^2+^ and ROS, PM phospholipids centrally regulate plant HS-sensing [40]. Notwithstanding their structural roles as constituents of the PM, phospholipids, viz., phosphatidylinositol 4,5-biphosphate (PIP2), phosphatidic acid (PA) and their metabolic enzymes phospholipase C and D (PLC/PLD) connect stress-signal perception to cellular responses [40,42]. Taken together, upon elicitation, Ca^2+^, ROS and lipid sensors trigger different signal transduction modules that activate HS response, which functions to restore cellular homeostasis [16].

Other key components of the HS signalling cascades are protein kinases (PKs) and protein phosphatases (PPs) that mediate protein phosphorylation and dephosphorylation, respectively. These include Ca^2+^-regulating proteins (calmodulins, CaMs), calmodulin-like proteins (CMLs), CDPKs (also known as CaM-binding protein kinases, CBKs), calcineurin B-like proteins (CBLs), CIPKs, and mitogen-activated protein kinases (MAPKs) [44,50]. CaMs are the most important and evolutionary conserved primary cytosilic Ca^2+^ sensors perpetuating intracellular Ca^2+^ homeostasis [49]. CaMs and CMLs contain helix-loop-helix EF-hand domains that regulate their downstream targets based on Ca^2+^ oscillations. CaM3 acts as a converter of the Ca^2+^ signals through interaction with CBK3 and PP7, which activates Heat Shock Transcription Factors (HSFs) that subsequently tune the expression of downstream HSPs [51]. HS-induced expression of a rice gene *OsCaM1-1* in Arabidopsis evoked the upregulation of HS-responsive downstream factors CBK3, HSFs and HSPs, consequently enhancing the HS tolerance of the transgenic Arabidopsis [52]. In addition, the gain-of-function mutants for *AtCBK3* gene in Arabidopsis exhibited improved HS tolerance, while the loss-of-function mutants showed HS sensitivity, revealing the crucial role of CBK3 in enhancing HS tolerance [51].

**Figure 1:**
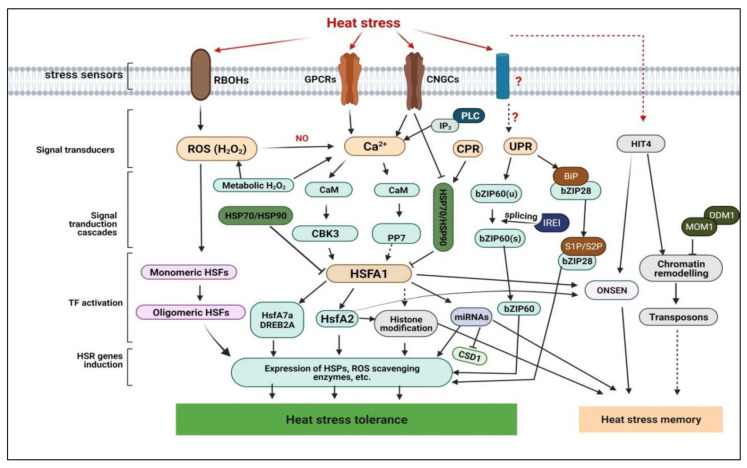
Molecular regulatory networks underpinning plant HS response and HS memory. HS alters plasma membrane (PM) fluidity, thereby initiating a lipid signalling pathway and inducting Ca^2+^ channels. Other PM-embedded receptors, such as the CNGCs, RBOHs, etc., perceive HS signals. Elevated cytosolic Ca^2+^ ions evoke the Ca^2+^ signalling pathways, mediated by the calmodulins (CaMs), CBKs or CDPKs (see text for details). The CaM interacts with CBK3 and CaM-binding protein phosphatase 7 (PP7) to activate HSFA1 by phosphorylation and enhanced regulation, respectively. The HSFA1 regulates the induction of several other HSFs, including HSFA2, dehydration-responsive element-binding 2A (DREB2A), HSFA7s, and micro-RNAs (miRNAs, e.g., miR398) that initiate the expression of HSPs and other heat stress-inducible genes, which eventually orchestrates physiological responses precipitating thermotolerance acquisition. Meanwhile, the ROS (H_2_O_2_) sensed by the RBOHs, together with nitric oxide (NO) and metabolic H_2_0_2_ generated from chloroplasts, mitochondria, etc., trigger ROS build-up, which initiates the ROS signalling pathway, and ultimately alters the expression of HSPs and other HS-inducible genes, via the activation of monomeric HSFs (inactive form in the cytoplasm), which yield oligomeric HSFs (active form which is translocated into the nucleus) to activate HSP gene expression. The PM-sensed HS signals, or ROS burst emanating from the endoplasmic reticulum (ER)-generated HS also activate the cytoplasmic protein response (CPR) and unfolded protein response (UPR) mechanisms. The UPR network is largely mediated by the inositol-requiring protein-1 (IRE1)-*bZIP60* mRNA and the site-1/site-2 proteases (S1P/S2P)-bZIP28 pathways. Upon ER stress, IRE1 catalyzes the splicing of the *bZIP60* mRNA into a truncated bZIP60 (s) variant that encodes a bZIP60 protein lacking a transmembrane domain, which allows for its translocation from the ER into the nucleus, where it will activate HS-responsive genes expression. Under benign conditions, BiP (an ER-embedded chaperone) maintains *b*ZIP28 at the ER. However, ER stress causes the relocation of bZIP28 to the Golgi apparatus, where it undergoes proteolytic processing, consequently triggering nuclear relocation of its cytoplasm-facing domain, where it ultimately evokes HS-responsive genes expression. Meanwhile, histone modification (HM) and chromatin remodelling (CR) also stimulate HS-inducible gene expression. In addition, HM, CR, ONSEN and some transposons modulate plant HS memory (HSM). *Heat-intolerant 4* (*HIT4*) is a central regulator of CR in response to HS and facilitates nucleosome dispersion leading to the release of transcriptional gene silencing. HSFA2-derived HM offers transient HSM that lasts for a few days and facilitates rapid gene reactivation, whereas CR, ONSEN, and transposons-mediated HSM last for a longer period and provides long-term adaptation to plant HS. **Note:** Dashed lines and question marks signify links yet to be confirmed. **Other abbreviations mentioned in the diagram:** GPRCRs, G-protein coupled receptors; PLC, phospholipase C; IP3, inositol triphosphate; CSD1, copper/zinc superoxide dismutase 1 (CSD1); DDM1, DNA methylation 1; MOM1, Morpheus’ molecule 1.

CBKs also interact with HSFA1 and alter the DNA-binding ability of HSFA1, consequently modulating the expression of target HS-responsive genes [18]. Moreover, PP7 is hypothesized to dephosphorylate HSFA1, which then tunes HSFA1 functioning during HS, suggesting that HSFA1 is partly regulated by phosphorylation/dephosphorylation of CBK3/PP7 [49]. The coupling of Ca^2+^ to the CaMLD of CDPKs activates their kinase activity and initiates the phosphorylation of target-signalling networks. Therefore, CDPKs perform a dual function of detecting Ca^2+^ signals and responding through phosphorylation actions in opposition to HS signals [18]. At the protein phosphorylation/dephosphorylation cascade terminals, PKs or PPs activate or suppress HSFs and other TFs, respectively. These TFs become specifically coupled to cis-regulatory elements in the promoters of HS-responsive genes, eventually evoking their transcription [50,53]. The molecular regulatory network underpinning plant HS response is displayed in Figure 1.

#### 3.1.2. Essential Molecular Regulators of Thermo-/Photo-Sensing and HS Signaling

Over the last decade, accruing evidence has revealed the tight link between temperature and light sensing and signalling in plants [21]; both factors share similar receptors, molecular regulators and signalling pathways [54,55]. Unlocking this crosstalk between light and temperature signalling in plants has been critical in revealing HS sensing and signalling [21]. Phytochrome Interacting Factors (PIFs), evolutionarily conserved from liverworts to angiosperms and part of the basic helix–loop–helix (bHLH) TF subfamily, interact specifically with the Pfr conformer of phy photoreceptors via dimerization and DNA-binding capacity enhancement of their conserved bHLH domain [56]. In particular, PIF4 is a core **thermomophogenesis**-signalling hub on which various temperature signalling modules converge [57,58]. Moreover, PIF4 enhances the expression of auxin (*YUC8*, *IAA19*, *IAA29*, *SAURs*, etc.) and brassinosteroid (BR) biosynthesis-related (e.g., *COG1*) genes [58,59], suggesting that PIF4 induces thermomophogenesis in a BR-dependent manner [21]. Furthermore, mild HS decreases the evening complex (EC) binding to its target genes, leading to increased *PIF4* expression [60].

Phytochrome B (PhyB) has been shown to integrate light and temperature signals; phyB is a highly temperature-sensitive photosensor that has two alternative conformations, the active form (Pfr) and the inactive form (Pr). The R light promotes a shift of phyB to the Pfr conformation, whereas FR light promotes the reversion of phyB to its Pr conformation [54]. Moreover, the change from the Pfr to Pr form can occur naturally and is temperature-dependent. This temperature-sensitive dark reversion of the active Pfr to the inactive Pr conformation facilitates mild HS perception by the phyB photosensor [21,54]. Crucially, since the active Pfr conformation of phyB and cooler temperatures promote degradation and inhibition of PIF4 functioning, the reversion of phyB to its inactive Pr confirmation due to mild HS causes the rapid extrusion of nuclear speckles that releases PIF4 inhibition. Consequently, the process of thermomophogenesis will be initiated [54,57].

Early Flowering 3 (ELF3) constitutes one of the three components of the EC that control the circadian clock [60]. ELF3 contains a prion-like domain (PrLD) with a high proportion of glutamine residues [61], and its temperature sensing mechanism is based on the conditional formation of condensed liquid phases within a bulk dilute phase necessitated by the coalescence of proteins through their PrLDs [40]. In addition to the regulation of the circadian clock, EC coordinates endogenous and environmental signals in plants [62] and represses the expression of thermomophogenesis enhancing factors such as PIF4, limiting the period of temperature-induced growth [40]. However, the temperature-dependent phase separation of ELF3 into inactive liquid droplets (condensates) at mild temperatures contributes to thermosensing and thermomophogenesis [21,61].

Similar to PIF4, in response to elevated ambient temperature, PIF7 has been shown to promote thermomophogenesis in Arabidopsis, by attaching to the promoters of auxin biosynthetic or signalling (ABS) genes, consequently inducing their expression [63]. Additionally, PIF7 and PIF4 are thought to depend on each other, possibly by forming heterodimers [63]. Further, mild elevated HS is suggested to rapidly enhance PIF7 protein accumulation, potentially contributing to thermomorphogenic response. The PIF7 transcript undergoes enhanced translation in response to mild HS, mediated by the formation of an RNA hairpin within its 5’ untranslated region, which adopts an alternative conformation at a higher temperature, resulting in increased PIF7 protein synthesis [64]. The accumulation of the PIF7 protein directly evokes a thermomophogenic response by inducing the expression of auxin biosynthetic gene *YUCCA8* [64]. However, it remains to be investigated how these receptors behave and interact in response to light or HS in cereal plants at the R stage.

### 3.2. Short-Term Heat Stress Escape and Avoidance Mechanisms

#### 3.2.1. Thermomophogenesis

Plants respond to temperature variations by instituting short-term avoidance and acclimation mechanisms such as leaf re-orientation and acceleration of transpiration to enhance cooling [1]. Mildly elevated temperatures can trigger considerable expressions of HS responsive genes, consequently resulting in obvious plant morphological and developmental alterations, such as leaf hyponasty, petiole elongation, reduced stomatal density and accelerated flowering. This response is termed thermomophogenesis [12,21] (Box 1).

Several crop plants (including some wheat and rice lines [65,66]) tend to mature early under HS, which leads to small yield losses, and this is an essential heat escape mechanism [1,34]. Another effective heat-escape strategy that helps crops, such as rice and wheat, to minimise HS damage during anthesis involves shifting the peak flower opening time, either naturally or by genetic means, towards the cooler hours of the day. This is termed the time-of-day-of-flowering (ToDF) mechanism [11]. For instance, HS exposure during anthesis can lead to sterility in rice. Therefore, some HS-resilient rice genotypes have adopted the early morning flowering (EMF) trait that assists plants in escaping HS damage [66]. In spring wheat, some HS-tolerant cultivars employ both EMF and late evening flowering (LEF) strategies to minimise high-temperature exposure and HS damage [67]. This avoidance mechanism allows plants to complete the fertilization process before the onset of detrimental (elevated) temperatures that may cause sterility [12,66]. Other short-term acclimation and long-term adaptation mechanisms are outlined (Table 1).

#### 3.2.2. Cell Plasma Membrane Lipid Composition Adjustment

The PM is the most HS-sensitive macromolecular structure in the cell [68]. As such, upon sensing HS, PM bilayer integrity safeguards are instituted through heat-induced biophysical alterations in PM properties, including increasing fatty acids disorganization, rotational movement and lateral diffusion, as well as decreasing the lipid headgroup-packing density [40]. These parameters constitute different aspects of the membrane fluidity, and these PM fluidity alterations affect the folding, mobility and functioning of the PM proteins. While these alterations can have detrimental effects on cell functions, even at moderate scales, they can serve as a basis for HS-sensing and acclimation [19,40].

Whereas increased levels of unsaturated fatty acids in lipid membranes help plants to survive in cold temperatures, increased levels of saturated fatty acids ensure survival under high-temperature conditions [68]. FATTY ACID DESATURASES (FADs) enzymes critically regulate the levels of these bipolar fatty acids, thereby sustaining optimum levels of cell membrane fluidity under an altered thermal state [49]. FADs are disorganized by elevated temperatures, resulting in increased saturation level that helps confer thermotolerance in plants [68]. HS-induced membrane fluidity alterations may activate PM-bound PPs and PKs, evoking rapid increases of PA and PIP2, which are vital in stress-signal transductions [41]. Among the eight FAD family members (*FAD*1 to *FAD8*) in Arabidopsis, *FAD2* and *FAD3* are endoplasmic reticulum-localized, while the rest are PM-embedded [68]. PM-embedded FAD members *FAD7* and *FAD8* are the most important in HS response.

Plant plastidial ω-3 *FAD7* catalyses the synthesis of C16 and C18 trienoic fatty acids, essential for cell membrane fluidity modulated cold temperature tolerance [69]. Silencing of the gene encoding chloroplastic *ω-3 FAD7* (which synthesizes trienoic fatty acids essential for cold temperature tolerance) resulted in decreased levels of trienoic fatty acids and enhanced high-temperature acclimation in *FAD7*-silenced transgenic tobacco plants [70]. In the *FAD7 FAD8*-deficient Arabidopsis line, dienoic fatty acids (16:2 and 18:2) were elevated, while trienoic fatty acid (18:3) decreased under high-temperature conditions, triggering thermotolerance [71]. Similarly, increased 18:2 dienoic fatty acids and reduced 18:3 trienoic fatty acids in *FAD7 FA8* rice mutant plants under high-temperature-induced thermotolerance [72]. Furthermore, since the wheat *FAD7* gene primarily functions in the accumulation of trienoic fatty acid 18:3 in the plastidial membrane, knocking down the *FAD7* gene may alter the fatty acids levels/ratio and help achieve HS tolerance in wheat [49]. Thus, FADs play critical roles in plant HS responses.

### 3.3. Heat Stress Tolerance Mechanisms

Mechanisms that promote the maintenance of essential plant functions and ensure plant productivity under HS conditions contribute to HS tolerance. These mechanisms enhance plant evolutionary adaptation under a HS environment [4]. Component strategies include activation of the antioxidant defense system, transcriptional regulation of HS response, and initiation of HS responsive genes, among others (Table 1).

#### 3.3.1. Antioxidant Defense System

In response to HS-induced ROS overproduction, plants activate their intricate non-enzymatic (including proline, Pro; spermidine, Spd; glutathione, tocopherols, etc.) and enzymatic antioxidant (superoxide dismutase, SOD; catalase, CAT; glutathione S-transferase, GST; glutathione reductase, GR; peroxidases (guaiacol and ascorbate), etc.) systems [41]. Amplified antioxidant enzyme activities and decreased ROS build-up in developing anthers contributed to HS tolerance and sustained grain yield in wheat [73]. Transgenic rice plants overexpressing (OE) *OsRab7* gene showed greater survival rate, Pro content, relative water content and antioxidant enzyme (SOD, CAT, APX and POD) activities, but decreased H_2_O_2_ and malondialdehyde (MDA) levels, as compared to their Wt counterparts [74]. Moreover, *OsRab7* OE plants showed upregulated expression of four genes encoding ROS-scavenging enzymes (*OsCATA*, *OsCATB*, *OsAPX2*, and *OsSOD-Cu/Zn*) and improved grain yield, suggesting that *OsRab7* enhances drought and HS tolerance in rice by modulating antioxidants accumulation and expression of ROS-scavenging and stress-responsive genes [74]. Furthermore, *ZmHs06* OE transgenic Arabidopsis plants exhibited higher SOD, POD, and CAT activities, but lower relative electrical conductivity and MDA content than the Wt controls under HS and drought stress conditions [75].

Maize plants OE *ZmCDPK7* displayed greater HS tolerance and antioxidant enzyme activity but lowered the H_2_O_2_ and MDA contents more than the Wt plants under HS conditions, while *ZmCDPK7*-knockdown plants exhibited the opposite patterns [76]. Additionally, *ZmCDPK7* was shown to interact with the sHSP17.4 and phosphorylate sHSP17.4 at Ser-44 and RBOH at Ser-99 and upregulate their expression. Further, RBOH inhibition experiments revealed that abscisic acid (ABA)-induced *ZmCDPK7* functions both down- and upstream of RBOH and is involved in maize HS tolerance through mediating phosphorylation of sHSP17.4, which might be essential for its chaperone activities, and as physiological results suggested, via increased ROS scavenging activities [76].

Meanwhile, spermidine (Spd) decelerated superoxide anions accumulation rate but increased protective enzymes SOD and CAT activities under non-HS and HS conditions. Additionally, Spd enhanced free polyamine content and expression of polyamine biosynthesis enzyme gene *OsADC1* (arginine decarboxylase 1) in rice spikelets under HS before heading, suggesting the role of Spd in the antioxidant defense system [77]. In addition, OE of *OsProDH* gene, encoding for proline dehydrogenase, decreased Pro content, while *OsProDH* knockout mutants exhibited elevated Pro levels as compared to Wt under high temperature in rice. The finding that *OsProDH* OE lines were more HS-sensitive while the *OsProDH* knockout mutants were more HS-resistant compared to their Wt counterpart suggested that *OsProDH* negatively regulates thermotolerance in rice by modulating Pro biosynthesis and ROS scavenging [78]. Furthermore, OE of *OsANN1* (ANNEXIN 1), a Ca^2+^-binding protein, in transgenic rice plants promoted SOD and CAT activities, which modulated H_2_O_2_ flux and redox homeostasis, suggesting the existence of a feedback mechanism between *OsANN1* and H_2_O_2_ accumulation under HS [79].

#### 3.3.2. Transcriptional Regulation of HS Response

Transcriptional regulation of HS response has been extensively detailed in recent excellent reviews [3,19,80]. TFs have been designated key transcriptional activators or molecular switches central to HS response [81]. Structurally, plant HSFs share a well-conserved structure comprising an N-terminal DNA binding domain that specifically binds to the HSEs, subsequently evoking HS-inducible gene induction [81]. In particular, HSFA1s function as the main transcriptional activators of other HS-responsive factors such as HsfA2, DREB2A, HsfA7s, etc. [82] (Figure 1). HSFA1s are upregulated by HS and HSFA1 activity is modulated by high temperatures [3], while HSP70 and HSP90 inhibit HSFA1 activity and nuclear localization under optimum temperature conditions; HS stimulates the dissociation of HSFA1 from HSP70 and HSP90, prompting HSFA1 activation [80].

Recently, HSFA1 has been shown to interact with BRASSINOSTEROID INSENSITIVE 1 EMS-SUPPRESSOR 1 (BES1), a TF subfamily possessing bHLH-like features, to enhance HS tolerance in Arabidopsis, with HSFA1 facilitating HS-induced de-phosphorylation, activation and HSE binding of BES1 in a BR-independent manner. This is partially mediated by ABA-repressed PP2C phosphatases [83]. Consequently, the BES1 evokes the expression of HSP70 and HSP90 genes via direct binding to HSEs, as facilitated by HSFA1s under HS conditions [83]. Overexpression (OE) of wheat HSF gene *TaHSFA6f* in Arabidopsis conferred improved tolerance to HS and other abiotic stresses [84]. Meanwhile, *TaHSFA2-10* OE transgenic Arabidopsis plants displayed enhanced HS tolerance as compared to Wt counterparts, as *TaHSFA2-10* regulated the binding of *AtHSPs* to HSEs, and their upregulation [85].

The endoplasmic reticulum (ER), as the primary site for lipids and protein biosynthesis, can suffer physiological or abiotic-factor-triggered stress. In response, the cell activates the adaptive unfolded protein response (UPR) mechanism [86]. The ER UPR safeguards protein homeostasis by elevating the ER’s protein folding, quality control and degradation capacities [86]. The misfolded and aggregated proteins are retrotranslocated into the cytosol where they are degraded by the ubiquitin-proteasome system known as the ER-associated degradation (ERAD) machinery [87]. Central to the ER UPR mechanism is the increased expression of genes encoding ER chaperones and components of the ERAD machinery [86,87]. Inositol Requiring Enzyme 1 (IRE1), which is an ER-embedded sensor protein, orchestrates ERUPR induction to rebalance the protein-folding homeostasis. In plants, IRE1 acts as an RNA splicing factor to activate the bZIP60 TF, via ER luminal stress-sensing-, cytoplasmic kinase-, and RNase domains [88]. Upon ER stress, IRE1 undergoes a conformational change, consequently splicing bZIP60 mRNA into a form that encodes a bZIP60 TF without a transmembrane domain [42,87,88]. The binding of unfolded proteins to the ER’s luminal domain evokes dimerization and induction of RNase activity that cuts the unspliced bZIP60(u) mRNA into spliced bZIP60(s) variant. Then, the spliced bZIP60(s) variant is translated, yielding an active bZIP60 TF protein, which is imported into the nucleus to activate stress-responsive genes [42,86] (Figure 1).

The cytosolic UPR (CUPR) mechanism, involving specific HSFs, especially HSFA2, functions to preserve cytosolic protein homeostasis [42]. In the CUPR, **heat shock** triggers HSFs, consequently inducing an elevated expression of HSP genes [53]. Plant HSPs and sHSPs act as molecular chaperones that are recruited to damaged proteins to facilitate their repair or removal or prevent the irreversible aggregation of denaturing proteins [49,53,89]. For instance, HSP70, HSP90 and HSP100 genes exhibit upregulation to confer HS tolerance in barley at the R stage [90]. *OsHSP101* improves rice HS tolerance [91], while transgenic Arabidopsis plants OE HSP100 induce enhanced thermotolerance [92]. Additionally, transgenic Arabidopsis plants OE a HS-responsive sHSP wheat gene *TaHSP23.9* exhibited enhanced tolerance to heat and salt (H+S) stress, suggesting that *TaHSP23.9* functions as a chaperone to positively regulate plant responses to H+S stress [93]. Furthermore, the expression of maize HSF gene *ZmHsf06* enhanced the basal and acquired thermotolerance and drought-stress tolerance of transgenic Arabidopsis plants [75].

HSFA1s can also trigger HS-inducible gene expression via transactivation of other HSFs such as Dehydration-Responsive Element Binding Protein 2a (DREB2A), HSFA2s, HSFA3s, HSFA7s, HSFBs, etc. [3,53] (Figure 1). HSFA1 transactivation competence is facilitated by crosslinking with HSP70 and HSP90 under HS [82]. DREB2A, a member of the AP2/ERF (Apetala2/Ethylene-Responsive Element Binding Protein) TF family, is directly targeted by HSFA1 [19]. In Arabidopsis, DREB2A directly regulates HSFA3 transcription by forming a coactivator complex with Nuclear Factor Y, Subunit A2 (NF-YA2), NF-YB3, and DNA Polymerase II Subunit B3-1 (DPB3-1)/NF-YC10 which bind the promoters of HSFA3 and induct its expression [5,30,94]. HSFA3 dysfunctional (mutant) lines exhibited decreased thermotolerance, demonstrating the role of DREB2A in transcriptionally regulating HSFA3 to enhance plant thermotolerance [94]. Essentially, DREB2A integrates HS- and drought stress responses by evoking the respective set of genes. For instance, transgenic Arabidopsis plants OE maize TF *ZmDREB2A* had improved tolerance to heat and drought (H+D) stresses [95].

Other TF families that regulate heat-responsive genes include the WRKY (possessing a highly conserved WRKYGQK motif at the N-terminus and a zinc-finger motif at the C-terminus), NAC (**NAM**, **ATAF1/2**, and **CUC2** domains containing proteins), MYB (myeloblastosis; characterized by highly conserved N-terminal MYB DNA-binding domain repeats), and bZIP (basic leucine zipper) [96]. Constitutive expression of *OsWRKY11* under the control of HSP101 promoter enhanced tolerance to H+D stress in transgenic rice plants [97]. *SNAC3* OE rice plants showed enhanced tolerance to H+D and oxidative stresses, while *SNAC3*-suppressed plants showed increased sensitivity to these stresses [98]. Additionally, *SNAC3* OE transgenic plants exhibited remarkably decreased levels of H_2_O_2_, MDA and relative electrolyte leakage than the WT plants under HS conditions, suggesting that *SNAC3* confers HS tolerance through modulation of ROS homeostasis in rice [98].

HS induces the expression of wheat NAC TF gene *TaNAC2L*, and *TaNAC2L* OE Arabidopsis plants exhibited improved acquired thermotolerance as compared to Wt plants [99]. Compared to their WT counterparts, *bZIP17* knockout mutant Arabidopsis plants showed sensitivity to HS at the R stage, demonstrating that *AtbZIP17* is essential for RSHS tolerance, by regulating HS-responsive gene expression in flowers [100]. Taken together, several HSFs, HSEs and HSPs constitute the complex GRN involved in the transcriptional regulation of HS response.

#### 3.3.3. Phytohormonal Regulation of HS Response

Phytohormones indispensably integrate HS signals by activating diverse transduction pathways, which often involve PPs and PKs [18]. Mutants deficient in ABA metabolism and signaling exhibited greater susceptibility to combined H+S stresses as compared to Wt Arabidopsis plants, suggesting that ABA is required for plant acclimation to H+S combination [101]. Additionally, ABA signalling evokes the ABA-mediated HS response in Arabidopsis through activation of downstream effector HSFA6b [102]. More recently, the inhibition of NO has been shown to significantly reduce ABA-induced osmolytes and antioxidant metabolism in wheat, suggesting that ABA function in HS response is NO-dependent; NO and ABA mediates HS tolerance through regulation of osmolytes and antioxidants [103].

Brassinosteroids (BRs) increase the expression of several HSPs, thereby enhancing HS tolerance [104]. In barley, Wt plants exhibited increased expression in HSP genes, while their BR mutant counterparts showed a reduced HSP gene expression under HS conditions, suggesting the role of BRs in HS acclimation [104]. Meanwhile, BR-mediated H+S stress tolerance in Arabidopsis has pointed to crosstalk that possibly exists amongst BR with ABA, salicylic acid (SA), and ethylene (ET) signalling pathways through these hormones sharing similar transcriptional targets [105]. Although ABA was shown to inhibit BR effects in plant stress responses, *NPR1* (*Nonexpressor of Pathogenesis-Related Genes 1*) was proposed as a vital component of the BR-mediated H+S tolerance, while several hormone-responsive genes were also BR-responsive [105].

Jasmonic acid (JA) and SA synergistically confer basal thermotolerance in Wt Arabidopsis [106]. The exogenous application of methyl jasmonate (MeJA) helped to mitigate HS effects in Wt Arabidopsis through plants accumulating a diverse range of jasmonates such as JA 12-oxophytodienoic acid (OPDA), MeJA and JA-isoleucine (JA-Ile) under HS conditions, which activated the antioxidant defense system. Further, the mutant analysis showed that the Constitutive Expressor of PR1 Protein (cpr5-1) mutant plants exhibited constitutive induction of JA, SA and ET signalling pathways and enhanced HS tolerance [106]. SA reduces HS-induced membrane damage and regulates antioxidant enzyme (SOD, CAT, POD, etc.) activities [107]. In wheat, SA alleviated the HS effects on photosynthesis by enhancing Pro biosynthesis via increased γ-glutamyl kinase and decreased proline oxidase (PROX) activity, resulting in maintained photosynthetic activity under HS conditions [108]. Suggestively, SA interacts with Pro metabolism and ET formation to alleviate the HS effects on photosynthesis in wheat [108].

**Figure 2:**
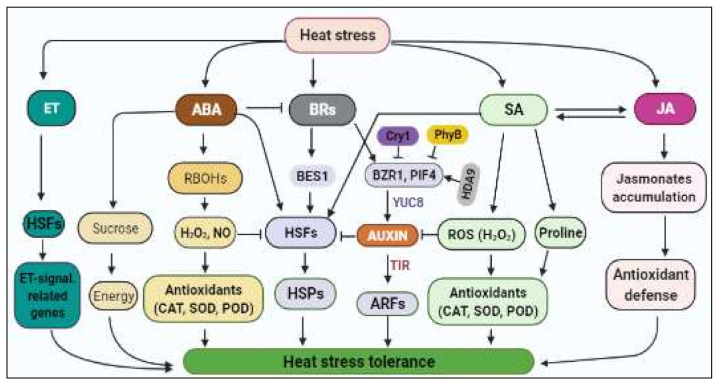
Phytohormonal signalling networks mediating plant HS response. The ABA signalling pathway evokes the ABA-mediated HS response through activation of downstream HSFs (e.g., HSFA6b), which then stimulate HSPs expression. The expressed HSPs invigorate photosynthesis and protein homeostasis under HS conditions, consequently leading to improved HS tolerance. Additionally, ABA mediates HS tolerance in a nitric-oxide (NO)-dependent manner, whereby the respiratory burst oxidase homologs (RBOHs), H_2_O_2_ and NO-transduced HS signals regulate osmolytes accumulation and antioxidant enzyme activities. In addition, ABA modulates the levels of carbohydrates and energy status via accelerated transport and enhanced metabolism of sucrose to enhance plant HS tolerance. Meanwhile, sucrose alone may act as a regulatory signal and/or provide energy, thereby contributing to HS tolerance. However, ABA has been shown to inhibit brassinosteroids (BRs) functioning in HS response. BRs activate HSPs through BR-controlled transcription factors such as Brassinosteroid Insensitive 1 (BRI1), EMS-Suppressor 1 (BES1), Brassinazole Resistant 1 (BRZ1) and phytochrome interacting factors (PIFs, e.g., PIF4, PIF7, etc.). BES1 may also evoke the heat shock response pathway mediated by ABA-repressed PP2 C-type phosphatases. BZR1 and PIFs, as regulated by BRs, play critical roles in auxin-mediated thermomophogenesis, by regulating the expression of auxin-biosynthesis genes, via auxin-responsive factors (ARFs). The YUCCA8 (YUC8) and Transport Inhibitor Response 1 (TIR) also play central regulatory roles in this BR-Auxin signalling pathway. Meanwhile, the chromatin-modifying enzyme Histone Deacetylase 9 (HDA9) mediates histone deacetylation at YUC8 nucleosomes to promote H2A.Z depletion, which then allows for the binding of the transcriptional regulator, e.g., PIF4 to YUC8 promoter. Conversely, light/thermo- receptors Crytochrome 1 (CRY1) and Phytochrome B (PhyB) suppress the HS-induced activation of PIFs and the expression of auxin-responsive genes. In addition, BR-mediated HS tolerance has pointed to a crosstalk that possibly exists among BRs with ABA and SA signalling pathways, through these hormones sharing similar transcriptional targets. Salicylic acid (SA) is known to trigger HSFs, which then activate HSPs to restore protein homeostasis, thereby inducing HS tolerance. Additionally, SA increases reactive oxygen species (especially H_2_O_2_) accumulation, which triggers the activation of antioxidant enzymes, consequently improving HS tolerance. Furthermore, SA enhances proline biosynthesis resulting in increased antioxidant enzyme activities and maintenance of photosynthetic activity under HS conditions. Exogenously applied jasmonic acid (JA) increases the accumulation of jasmonates such as methyl jasmonate, 12-oxophytodienoic acid and JA-isoleucine (JA-Ile), which activate the antioxidant defense system, consequently leading to HS tolerance. Meanwhile, JA and SA have exhibited cross-linkage, while JA and ET are antagonistic in their HS response regulation. Ethylene (ET)-mediated signalling involves ET modulating the transcript levels of HSFs (e.g., *HSFA1a* and *HSFA2a*, *b*, etc., which then stimulate ET-signalling-related genes (e.g., *Ethylene Insensitive 2*, *Ethylene Insensitive*-*Like 1*, *Ethylene Insensitive*-*Like* 2, etc.). Meanwhile, auxin plays a critical role in HS-induced thermomorphogenesis. **Note:** Arrows depict positive regulation while lines with blunt ends indicate inhibition or negative regulation. **Other abbreviations mentioned in the diagram:** HSFs, heat shock factors; SOD, superoxide dismutase; POD, peroxidases; CAT, catalase.

Ethylene-mediated signalling conferred HS tolerance by regulating transcript levels of HSFs (*HSFA1a* and *HSFA2a*, *b*, *c*, *d* and *e*) and ET-signalling-related genes (*Ethylene Insensitive 2*, *Ethylene Insensitive*-*Like 1*, and *Ethylene Insensitive*-*Like* 2) in rice plants under HS conditions, revealing the involvement of ET, HSFs and ET-signalling-related genes in a complex GRN mediating HS tolerance [109]. In addition, auxin has been shown to play a crucial role in HS-induced thermomorphogenesis in plants [18]. Taken together, phytohormones crosstalk assist in the regulation of plant HS responses (Figure 2).

## 4. Epigenetic Mechanisms, PTMs and Small RNAs in HS Response Regulation

Mounting evidence has uncovered the central role epigenetic mechanisms (EMs), PTMs and sRNAs play in plant HS response and adaptation [5,21,110]. Epigenetic modifications such as chromatin remodelling, DNA and histone methylation, RNA-mediated DNA methylation (RdDM), etc.; PTMs, including phosphorylation, SUMOylation, ubiquitination, etc.; and sRNAs, regulate HS-responsive genes expression [5,111]. EMs transcriptionally and post-transcriptionally modulate HS-responsive genes by altering the chromatin status of these genes [110]. Additionally, EMs are critical in the formation of epigenetic stress memory that can be transferred to the offspring of pre-stress-exposed plants [111]. The histone variant H2A.Z participates in several chromosomal processes such as DNA repair, transcriptional regulation and thermosensory responses. H2A.Z shows preferential deposition and enrichment in the +1 nucleosome after the transcription start site (TSS), consequently repressing gene expression [112]. However, HS causes the exclusion of H2A.Z from the +1 nucleosome on the promoter of the PIF4, consequently permitting chromatin accessibility and PIF4 binding to the G-box promoter element to evoke the induction of auxin-responsive and thermomophogenesis related genes such as *Indole Acetic Acid-Induced Protein 29 (IAA29)* [111].

Heterochromatic marks, including DNA methylation and H3K9me2 (histone H3 lysine 9 dimethylation), exhibit inhibitory effects on downstream gene expression by blocking the promoter region. Fortunately, HS triggers histone acetylation and methylation alterations that precipitate HS-responsive gene expression [80]. H3K4 hypermethylation is pivotal for HS-responsive gene expression and transcriptional HS memory, as governed by HSFA2 [113]. After HS exposure, HSP18, HSP22.0, HSP70 and APX2 accumulate H3K9 acetylation (H3K9Ac) and H3K4 trimethylation (H3K4me3), which play significant roles in hyper-induction of these HSP genes [5,110,113]. Equally, histone modification and DNA methylation via the RdDM pathway help plants acquire basal thermotolerance [114]. Chiefly, histone deacetylases (HDACs) and DNA methyltransferases (DMTs) orchestrate plant HS response [114]. HDA6, an RPD3-type deacetylase, is functionally associated with promoter silencing via the RdDM pathway. The HDA6-deficient mutants exhibited spurious RNA polymerase II (Pol II) transcription throughout the intergenic spaces, which, together with loss of deacetylase activity and maintenance of cytosine methylation of the corresponding genes, eliminated the repressive chromatin modifications, consequently inducting those target genes [115].

Further, the multifaceted role of DNA de/methylation in HS response has been revealed, largely via the activity of DMTs [116]. For instance, an investigation of HS effects on the expression of genes encoding key players in DNA methylation, including DMTs (*MET1*, *CMT3*, and *DRM2*), the largest subunits of PolIV and PolV (*NRPD1* and *NRPE1*, respectively), and DNA demethylase (*ROS1*), demonstrated that the coordinated upregulation of *DRM2, NRPD1* and *NRPE1* may orchestrate increased genome methylation under HS and PolIV and/or PolV may be required for the induction of *DRM2* expression under HS [116]. Meanwhile, HSFs are partly regulated by PTMs such as SUMOylation, phosphorylation/dephosphorylation, and PPIs [3,110]. For instance, SUMOylation of the HS TF AtHsFA2 is critical for HS responses and acquired thermotolerance in Arabidopsis [117].

sRNAs are a class of 18–30 nucleotides (nt), non-coding RNAs, which are synthesized endogenously from *microRNA (miRNA)* genes and found in both eukaryotes and prokaryotes [20]. In addition to their function in genome stability maintenance, sRNAs essentially modulate gene expression, either via direct cleavage of the target mRNA transcript or its degradation or inhibition at the translational level [118]. Based on diverse biogenesis pathways and modes of action, plant sRNAs are categorized into two major classes, viz., microRNAs (miRNAs) and small interfering RNAs (siRNAs) [20]. Several sRNAs have been characterized and implicated in modulating the expression of plant HS-responsive genes [119]. For instance, miR156, miR159, miR398, miR396, etc., regulate HS-responsive genes by modulating TFs activity in response to HS [80,118,119]. In Arabidopsis, the OE of miR156 creates HS memory in response to HS via miR156 targeting the *Squamosa-Promoter Binding-Like* (*SPL*) TFs, which subsequently downregulate the expression of HS-inducible genes [120]. In fact, the miR156 isoforms were highly induced under recurring HS (37 °C and 44 °C) conditions and enhanced the sustained expression of HS-responsive genes, suggesting that miR156 is functionally important for HS memory [120]. Meanwhile, down-regulation of miR159 under HS modulates MYB TFs. Specifically, miR159c and miR165b target the peroxidases, *Phenylalanine Ammonialyase* (*PAL*), RING-type E3 ubiquitin transferase, etc., which induce HS tolerance by promoting antioxidant metabolism and enhanced HSPs induction or degradation of suppressor proteins of HS-responsive genes [119,121].

Among the few HS-responsive siRNAs that have been studied in crop plants to date, Arabidopsis *trans*-acting small interfering RNAs (tasiRNAs) from the *trans*-acting siRNA 1 (*TAS1*) locus target Heat-Induced TAS1 Target 1/2 (HTT1/2) proteins that accumulate in response to HS [122]. In maize, the knockout of *dicer-like 5* (*Dcl5*), a reproductive-stage gene responsible for precise slicing to generate diverse 24-nt phased small interfering RNAs (phasiRNAs) in meiotic anthers of different monocots, conferred male sterility in null mutants (with complete loss of 24-nt phasiRNAs) under high-temperature conditions, suggesting that *Dcl5* and 24-nt phasiRNAs are critical for fertility under HS conditions for optimal yield [123].

Meanwhile, plants under recurring HS display transgenerational memory, as mediated by transposons [20]. In Arabidopsis, HS activated a copia-like retrotransposon, *ONSEN*, and the activated *ONSEN* are not only transcribed but also transgenerationally transposed in heat-stressed plants deficient in siRNAs [124]. Additionally, *ONSEN* are preferentially inserted within genes; HS activation and the insertion of *ONSEN* within or close to genes conferred heat responsiveness to the flanking genes, suggesting that *ONSEN* insertion may trigger gene network modification, possibly by targeting HSFA1 and HSFA2 (Figure 1) and play a pivotal role in transgenerational HS memory [5,124]. Taken together, EMs, PTMs and sRNAs play vital roles in HS response regulation at translational and post-translational levels, as well as HS memory maintenance, which helps plants survive HS conditions.

## 5. Strategies for Reproductive-Stage HS Tolerance Improvement

Efforts to invigorate HS tolerance in cereal crops encompass crop breeding, genomics, and omics approaches, as well as agronomic interventions; these strategies are discussed.

### 5.1. Genetics and Breeding Approaches

#### 5.1.1. QTL Mapping

Crop HS tolerance is a complex, quantitative and polygenic trait, whose enhancement is primarily by genetic improvement. Thus, researchers have spent the last three decades untangling the genetic architecture of plant thermotolerance [7,125,126]. Essentially, precise evaluation of the extent of variation of HS tolerance trait, selection of superior genotypes, and successful transfer of HS tolerance-related traits into specific elite cultivars remain the pillars to crop HS-tolerance breeding [30,127]. Conventional mapping methods, especially QTL mapping (or linkage mapping, LM), which is based on the genetic linkage of a quantitative trait with the molecular marker within a bi-parental segregation population (e.g., recombinant inbred lines, RILs), have been effectively used to identify crop HS tolerance-related QTLs and genomic regions, as well as genes underlying those traits [27,44,125,126,127,128]. The physiological traits essential for RSHS tolerance include stay-green or delayed leaf senescence, canopy temperature depression (CTD), membrane thermostability, improved flag leaf stomatal conductance and photosynthetic rate, chlorophyll fluorescence (F_v_/F_m_), enhanced grain filling rate and duration, number of fertile spikelets, and grain yield [34,128,129,130].

A significant positive correlation exists between the stay-green trait (SGT) and higher grain yield in cereal crop genotypes expressing this trait under drought and/or HS [131]. In wheat, for example, a stay-green QTL (*44 loci*) of the mapping population “Seri/Babax” grown under HS environment exhibited a strong positive link with HS tolerance, grain filling rate and grain yield [132]. The SGT-harbouring genotype maintains higher photosynthetic and grain filling competencies under HS conditions via deferred expression of chlorophyll degradation-related genes in winter wheat [133]. In barley, composite interval mapping identified ten QTLs for SG, among which six (including *HSPFLQ1*, *HSPFLQ2*, *HGSQ*, etc.) were HS-associated and four (including *WGSQ*, *WGFL1Q1*, etc.) were drought-stress-related [134]. None of the ten identified QTLs were co-located with previously reported barley stress-response QTLs and were therefore designated novel barley stress response QTLs [134]. The SGT displayed a significant association with canopy temperature depression (CTD) and yield traits under HS. CTD, defined as the deviation of plant canopy temperature from the ambient temperature [135], correlates well with plant water status and grain yield and is a vital indicator of a plant’s transpiration cooling capability under abiotic stresses [136]. RSHS-associated QTLs have been identified for CTD and yield-related traits [129].

Thylakoid membrane stability is highly associated with the HS tolerance capacity of plants [137]. Therefore, screening several genotypes for heat tolerance using membrane thermo-stability is more feasible. Using three bi-parental F_2_ mapping populations, 3 significant QTLs and 12 potential candidate genes linked to *F*_v_/*F*_m_ for enhanced photosynthetic efficiency under HS (40 °C for 3 days) during anthesis were detected in wheat [138]. Inclusive composite interval mapping showed that among these HS-associated QTLs, two (*QHst.cph-3B.1* and *QHst.cph-3B.2* in population 1110 × 810) and one (*QHst.cph-1D* in population 1110 × 1039) were located on chr 3B and chr 1D, respectively. These QTLs explained about 13–35% of the phenotypic variation for *F*_v_/*F*_m_ [138]. *frk2* and *bglu26* (involved in carbohydrate metabolism), *ndhB2* and *psaC* (involved in photosynthetic light reaction), *BUD31/G10* related, and two genes encoding chloroplastic 3-isopropylmalate dehydrogenase 2, were among the potential key HS-responsive genes identified from the *QHst.cph-3B.1* and *QHst.cph-3B.2* regions [138].

Using phenotypes of spikelet fertility or seed setting rate under HS, several QTLs linked to RSHS tolerance have been dissected in rice [1]. For instance, eleven QTLs for HS tolerance at anthesis were identified, further verifying *qPSL^ht^4.1* at several temperature regimes [139]; *qPSL^ht^4.1* locus had been previously detected in other studies [1]. Another study identified 12 QTLs linked to HS tolerance at the booting stage, with one of the major-effect (*qHTB3-3*) positioned near *qTL3.4* and *RLPC3.1* [140]. More recently, a trait-specific QTL survey identified that a tolerant rice genotype NL44 harbour novel genomic regions for RSHS tolerance in rice [141]. Single marker analysis in NL44 revealed five minor QTLs, four for spikelet fertility under HS (*qhs2*, *qSF4*; *SSPF*4, *qPF4*; *qHTB10-2* and *qHTB11*) and two for stress tolerance index (STI)-spikelet fertility (*qSF4* and *qPSLht7*), of which one QTL was mapped for both the traits. However, these QTLs could only explain a very low level of total phenotypic variation in rice [141].

Meanwhile, McNellie et al. [142] evaluated two biparental RIL populations (B73 × NC350 and B73 × CML103) for leaf and tassel heat tolerance traits in maize. In B73 × NC350, two traits (tassel blasting and reduction in spikelet size) were scored at flowering and only a single QTL for tassel blasting was detected on chr 5, explaining 7.96% of phenotypic variance. However, no QTLs were detected for the reduction in spikelet size [142]. Other important RSHS-related QTLs have been reported in wheat [143]. Taken together, these identified QTLs and genes can serve as direct targets for genetic engineering and selection for HS tolerance improvement in cereals, and the now-readily-accessible genome sequencing data will facilitate the map-based cloning of major QTLs controlling HS tolerance. However, LM has several drawbacks, including requiring a relatively long time to generate RILs of the two contrasting-phenotype parents and dependence on genetic recombination and segregation during the establishment of mapping populations, which compromise its mapping resolution allele richness [144].

#### 5.1.2. GWAS

Recently, association mapping (AM) via GWAS, which is based on genetic linkage disequilibrium (LD) and makes use of natural variation and recombinants [144], has become a highly desirable, quick and efficient tool to dissect complex QTLs such as HS tolerance [145]. Through the exploitation of historical recombinant events in a large genotype pool, LD mapping offers a comparatively high-resolution power and investigates multiple alleles of a single locus [144]. The deployment of more advanced multi-founder or multi-parental populations, e.g., nested association mapping (NAM) and the multi-parent advanced generation intercross (MAGIC) populations, helps increase the amount of genetic variation and facilitate high-resolution mapping of complex QTLs in crops [7,146], including RSHS-associated QTLs, e.g., spikelet fertility in rice [147]. Thus, propelled by advances in high-throughput DNA variation discovery techniques and statistical analyses, GWAS has gained much usage in crop genetic research [144].

For instance, three GWAS methods were applied to detect QTLs for spikelet sterility (SPKST) and numerous secondary traits under high temperature (38 °C) during anthesis in a panel of 167 *indica* accessions genotyped with 13 162 SNPs [148]. Fourteen independent loci significantly linked to SPKST under at least two GWAS methods were identified, among which eight co-localized with QTLs previously reported for HS tolerance during the R stage, including the most documented *qHTSF4.1* which was consistently detected across different genetic backgrounds [148]. Candidate genes underlying these SPKST-associated-QTLs were identified, including *OsWAK43* and *OsWAK44* (WAK receptor-like protein kinases) for *q04.2* on chr 4, *Os04g29960* (heat shock protein DnaJ) for *q01.2* on chr 1, *Os12g41410* (receptor-like protein kinase homolog RK20-1) for *q12.1* on chr-12, and other HS-response-associated genes in rice [148].

In addition, a combination of QTL mapping and GWAS analysis (using a panel of 261 diverse RILs, 259 973 SNPs, and 8329 SNP markers) was used to understand the genetic basis of HS tolerance of seed-set in maize under field conditions [149]. In total, four QTLs (*qSSR5-1*, *qSSR5-2*, *qSSR5-3*, and *qSSR5-4*) and 17 genes associated with 42 significant SNPs related to thermotolerance of seed-set were identified [149]. Among the key HS-related genes identified included peroxidases and auxin-responsive GH3 family proteins, among others. Notably, four candidate genes were detected by both linkage mapping and GWAS analyses [149].

Moreover, using an *indica* rice diversity panel comprising 209 genotypes, the genetic architecture of high night temperature (HNT; 24 °C/29 °C, control/HNT treatments) tolerance during panicle initiation to physiological maturity periods was analysed in rice [150]. GWAS analysis revealed 38 genetic loci associated across treatments, 18 for control and 20 for HNT. In addition, 20 candidate marker-trait associations (MTAs) were identified for the yield and quality-related traits under HNT across experiments [150]. Crucially, several GWAS loci were in the genic region or close to previously reported genes controlling HNT tolerance, indicating that GWAS is an effective approach to identify putative genes or target regions for HNT tolerance in rice [150]. Other more recent studies on GWAS analysis of QTLs, QTL hotspots and candidate genes underlying RSHS tolerance are available for maize [145], rice [147] and wheat [151]. However, despite GWAS for HS tolerance attempts in cereals, the proposed candidate genes or their causative variations still remain largely unverified and unresolved.

#### 5.1.3. Genomic Selection and Modern Plant-Breeding Methods

Although numerous marker-assisted breeding approaches, such as MAS, marker-assisted recurrent selection (MARS), etc., can facilitate the selection of favourable alleles for desired traits in early generations, the need for marker identification and overestimation of marker effects with small PVE remain their major limitations [152]. To overcome these drawbacks and to accelerate the rate of genetic gains in crop breeding programs, novel and powerful genomic tools for selection have been advanced, including genomic selection (GS) [153]. Compared to traditional selection methods, GS exhibits greater potential to shorten breeding cycles through the rapid selection of superior genotypes. Additionally, it excludes the need to unearth QTLs related to target traits and the need for the collection of phenotypes of the breeding population, thereby reducing costs [152]. GS utilizes genome-wide estimated breeding values (GEBVs) and exploits genome-wide genotype-phenotype association, thereby capturing both major and minor gene effects [154]. Already, GS has greatly revolutionized genotypic performance prediction and selection of complex traits such as disease resistance, yield and quality in wheat [155], drought tolerance in maize [156], and several biotic and abiotic stresses in diverse crops [154]. With the increased use of GS in crop improvement programs and intensification of RSHS tolerance breeding in cereals, the development of HS tolerance improved cultivars will be expedited.

In addition to GS, modern and innovative plant-breeding methods such as speed breeding [157,158], doubled haploid-based breeding (DHB) [159], **haplotype**-based breeding [160], fast forward breeding [161], and genome-editing-based breeding [162] approaches can be harnessed in RSHS tolerance breeding. Speed breeding (SB) technology encompasses growing plant populations under controlled environments such as glasshouses and growth chambers to shorten the breeding cycles and accelerates crop research via rapid generation advancement, for instance, via single-seed descent (SSD), and can potentially be deployed to large-scale crop breeding programs [158]. The SB approach has successfully achieved up to six generations per year for bread wheat, durum wheat, barley, chickpea and pea crops as compared to 2–3 generations per year under normal field conditions [157].

Doubled haploids (DHs) [127,159] permit homozygosity in segregating populations to be achieved in a single generation as compared to 5–7 generations by traditional breeding methods, allowing for earlier selection of stable and suitable genotypes [163]. Therefore, DHB offers a time-saving advantage for the infusion of quantitative traits that are not amenable to ready selection in the early segregating generations due to conventional crosses and considerably reduces the size of populations required to discover the desired line [163]. DHs have already been deployed in crop improvement programs in cereals, including rice, maize, wheat and barley [159,163], e.g., for GWAS mapping of yield-related QTLs and MTAs for HS tolerance in maize [145]. Meanwhile, haplotype-based breeding (HBB) deals with the detection of superior haplotypes (see Box 1) and their subsequent deployment into crop breeding programs [160]. A targeted assemblage of haplotypes can potentially minimize the trade-offs of traditional allele introgression methods to incorporate genomic regions into different genetic backgrounds [161]. Already, the HBB approach has shown its utility in trait identification in crops such as rice, where superior haplotypes of 21 genes regulating grain yield and quality traits across 3000 genomes were identified [164].

More recently, the fast-forward breeding (FFB) concept was proposed [161]. FFB offers a strategy for integrating advanced genome sequencing, crop phenotyping and systems biology approaches with efficient QTL mapping methodologies, genomic prediction technologies (encompassing machine learning algorithms and artificial intelligence), and other emerging breeding methods such as optimal contribution selection (OCS) to enhance the genetic base of breeding programs while accelerating genetic gains [161]. In the cases where crops are recalcitrant to DH technology (as is the case with most cereals); or where DH responsive species suffer from linkage drag, require further recombination or are not amenable to the currently available tissue culture techniques, modified pedigree breeding methods such as SSD can be the best alternatives to achieve gene fixation and quicker generation cycling [161,163]. Taken collectively, the increased adoption of modern plant breeding approaches such as GS, DHB, SB and FFB in HS tolerance breeding programs, coupled with gene-editing techniques, could accelerate the creation of HS resilient cereal cultivars.

### 5.2. Biotechnological Intervention and the Role of CRISPR-Cas9

#### 5.2.1. Potential Target Genes for Transgenic Improvement of RSHS Tolerance

Transgenic approaches facilitate the transfer of desirable traits/genes to elite cultivars, bypassing the linkage drag obstacle, involving co-transference of unsolicited adjacent gene segments, and enabling the exploitation of genes not accessible via hybridization-based breeding [161,165]. Recombinant DNA technology permits for intra-species or inter-species transfer of genes/traits, thereby enabling the harnessing of superior traits both from improved cultivars or wild types. This is largely underpinned by *Agrobacterium tumefaciens*–mediated transformation because of its ability to transfer larger DNA fragments, its reproducibility, and simple operation amenability [165,166]. Consequently, several genes or proteins, including TFs, HSPs, antioxidant enzymes, etc., have been successfully cloned, and their OE enhanced HS tolerance in different cereals, including maize, rice, wheat, etc. [9,44,126,129]. For instance, maize phosphoenolpyruvate carboxylase gene *ZmPEPC* overexpressed in wheat improved photochemical and antioxidant enzyme activities, upregulated expression of photosynthesis-associated genes, deferred leaf senescence, altered contents of proline and other metabolites, and eventually improved HS tolerance [167].

In rice, OE of sHSP gene *OsHSP18.6* improved tolerance to HS and other abiotic stresses in transgenic plants [168]. Moreover, transgenic plants exhibited decreased MDA content but enhanced antioxidant enzymes (SOD and CAT) activities under H+D stress conditions. Further, the *OsHSP18.6* OE lines showed decreased sterile rates under HS as compared to Wt [168]. The transgenic Arabidopsis OE maize gene *ZmWRKY106* exhibited improved tolerance to D+H stresses as a result of *ZmWRKY16* modulating stress-related genes via the ABA signalling pathway and reducing ROS accumulation in transgenic plants through enhancement of antioxidant enzyme (POD, SOD, and CAT) activities under D+H stresses [169]. Therefore, the modulation of HSFs, HSPs and other genes/proteins can help us engineer HS tolerance in cereals [170] (Table 2).

#### 5.2.2. Candidate Metabolic Pathways for Transgenic RSHS Tolerance Improvement

Targeted manipulation (either via knockout or OE) of key enzymes regulating core pathways implicated in HS response can aid in bioengineering novel HS-tolerant cereal crop cultivars. These core metabolic pathways include the γ-aminobutyric acid (GABA) biosynthesis, starch biosynthesis and phenylpropanoid biosynthesis pathways, among others. For instance, GABA, synthesized by glutamate decarboxylase (GAD) and shows accumulation in response to abiotic stresses, crucially regulates metabolic responses to heat [178] or combined heat and light stresses [179]. Exogenous supply of GABA significantly enhanced HS tolerance in creeping bentgrass (*Agrostis stolonifera*), mainly by increasing the accumulation of osmoprotectants, enhancing photosynthesis efficiency, and osmotic adjustment maintenance, and metabolic homeostasis maintenance [179]. In addition, key microRNAs (vvi-miR845c, ama-miR156, novel-24223, etc.) have been identified as potential effectors of GABA-regulated HS tolerance [178]. Therefore, accruing a repertoire of HS tolerance genes/proteins and novel HS tolerance-linked microRNAs from diverse (cultivated and wild) species could ensure robust and broad-based HS tolerance in rationally designed cereal cultivars.

ADP glucose pyrophosphorylase (AGPase), one of the four key enzymes involved in starch biosynthesis, plays a significant role in starch synthesis and regulation in cereal endosperms [180], by catalyzing the production of ADP-glucose (the first key enzymatic step), and is a rate-limiting enzyme in the starch biosynthesis pathway [181]. The transgenic cereal plants’ OE-modified AGPase forms exhibited an improved rate of starch biosynthesis and higher final grain yield [181]. Therefore, the thermotolerant variants of AGPase may be harnessed to create cereal crop cultivars with enhanced productivity under HS.

Phenylalanine ammonia lyase (PAL) catalyzes the initial steps of this phenylpropanoid biosynthesis pathway, by directing the metabolic flux from the shikimate pathway to several branches of phenylpropanoid [182]. Secondary metabolites derived from this pathway can then act to provide plant stress tolerance, probably via antioxidant actions [183]. Furthermore, phenylpropanoid biosynthesis pathway has been shown to crosstalk with BRs and ROS signalling in abiotic stress responses [184]. Meanwhile, a comparative transcriptomic study using two genotypes revealed that lignin and flavonoid biosynthetic pathways play critical roles in rice HS tolerance during the reproductive (meiosis) stage [185]. The heat-tolerant genotype SDWG005 maintained a steady-state balance of metabolic processes and showed greater lignin deposition and flavonoid accumulation under HS than the heat-sensitive genotype MH101, which explained better thermotolerance in the heat-tolerant genotype [185]. In addition, the carbon and nitrogen metabolism (C+NM) pathway can also be targeted for bioengineering RSHS tolerance in cereals [186]. Weakened C+NMs under post-silking daytime HS (35 °C) have been found to diminish dry matter accumulation and grain yield in waxy maize, largely by limiting photoassimilate deposition, suggesting the C+NM pathway plays a role in HS tolerance [186].

Currently, the CRISPR-Cas9 (clustered regularly interspaced short palindromic repeats (CRISPR)-associated enzyme 9)-based genome-editing technique is revolutionizing the precise editing of targeted genes in plants and potentially circumvents several drawbacks of the transgenic transformation approaches [187]. For details on CRISPR-Cas9 and other genome-editing tools (GETs), see the most recent reviews [187,188]. The advantages of CRISPR-Cas9 over other GETs, such as TALENS and ZFNs, lie in its great precision, efficiency, broad applicability, fitness for concurrent multiplex editing of target sites, rapidity, cost-effectiveness, and user-friendliness in developing non-transgenic (non-GMO) crops [187,188]. Furthermore, multiplex editing of targeted gene sites will significantly speed up gene stacking for important traits [161]. However, to date, the CRISPR-Cas9 technique has largely been deployed for other abiotic stresses, particularly D+S, but not in terms of HS [188] and rarely in cereals. Hence, we envisage that as the CRISPR-Cas9 approach becomes routinely applied in crops, it will be extended to cereal HS tolerance improvement efforts as well, ultimately galvanizing the transgenic efforts and modern crop breeding methods to rapidly develop heat-tolerant cultivars in a swiftly changing climate.

### 5.3. Omics-Driven Approaches

Over the past decade, flourishing development in omics technologies has revolutionized the way plant biologists dissect diverse mechanisms underlying plant tolerance to different environmental stresses [189]. These omics (see Box 1) approaches have necessitated the identification of key differentially expressed genes (DEGs), proteins (DEPs) and metabolites (DEMs) underpinning plant biotic and abiotic stress tolerance, using contrasting (tolerant and sensitive) genotypes in comparative transcriptomic, proteomic and metabolomic studies, respectively [130,189]. With regard to HS tolerance, comparative transcriptomics [190] and proteomics [191,192] analyses have revealed HS-responsive genes and proteins, as well as mechanisms regulating thermotolerance at the R stage in cereals. For instance, a comparative transcriptome analysis of HS (at 40 °C) tolerance mechanisms at panicle development and spikelet formation stage in rice revealed 1688 HS-responsive genes exclusively expressed in the tolerant genotype and 1675 shared HS-responsive genes [190]. Among the tolerant genotype-related HS-responsive genes, the WRKY, HD-ZIP, and ERF transcription factors were more prominent and suggested to play a critical role in HS tolerance of the developing panicles via the promotion of plant hormones and signal transduction pathways [190]. More recently, a comparative transcriptome study revealed that post-pollination HS caused kernel abortion in maize, especially in a heat-sensitive cultivar, as a result of carbohydrate metabolic disorders [193]. HS decreased the RuBPCase activity by down-regulating *Zm0001d052595* and *Zm0001d004894* genes, which restricted photosynthesis and caused insufficient assimilate availability for the developing kernels [193].

A proteomic analysis at the early grain-filling stage of rice using two-dimensional gel electrophoresis (2-DE) revealed a total of 27 DEPs in rice grains, predominantly from the heat-tolerant genotype under HS, among which oxoglutarate and glutamine synthetase (OsUP16), involved in glutelin synthesis, were upregulated [191]. Moreover, an investigation of contrasting wheat cultivars‘ response to HS (35 °C/26 °C, day/night) during the grain-filling stage revealed that proteins related to photosynthesis, heat shock, signal transduction, and antioxidants were differentially accumulated and contributed to higher HS tolerance in the tolerant genotype compared to the sensitive genotype [192]. Taken together, the key HS-responsive proteins unearthed through such comparative proteomic studies may serve as biomarkers to identify or genetically engineer HS-tolerant cereal crop cultivars.

Meanwhile, comparative metabolomics analysis of different wheat genotypes exposed to post-anthesis HS (35 °C/28 °C, day/night) identified 64 known metabolites that accumulated due to HS treatment [194]. Among these key metabolites, L-arginine, L-tryptophan, L-histidine and leucine were significantly higher in tolerant genotypes under HS. Additionally, aminoacyl-tRNA biosynthesis and plant secondary metabolite biosynthesis pathways were most impacted by HS, suggesting their important role in post-anthesis HS tolerance in wheat [194]. These few examples highlight the critical role omics approaches can play in informing GAB, GS and site-directed nucleases targets for RSHS tolerance breeding in cereals.

### 5.4. Agronomic Options

Crop management practices (aimed at addressing the effect of biotic and abiotic factors) are often a component of the G × E × M equation of yield improvement. This means that, in principle, crop yield can be boosted through adjustments to the genotype (G), growth environment (E), and management practices (M) [7]. In particular, the impact of HS on cereals can be partially reduced through improved crop management options [11,195]. For instance, advancing the sowing date and demand-driven irrigations may safeguard H+D-induced yield losses in cereals [196]. Early planting may avoid terminal HS so that the grain-filling period may coincide with cooler temperatures in crops such as wheat and rice [26,34]. Proper plant nutrient management (quantity supply, timing, etc.) may contribute to the increased ability of crops to cope with moderate heat increases [7,26,27]. An exogenous supply of micronutrients such as zinc can help plants tolerate short periods of HS, e.g., in wheat and maize [196], while conservation agriculture can potentially preserve soil moisture and enhance plant water and nutrient use efficiencies, consequently stimulating plant growth and yield under heat or H+D conditions [26,27,34].

Meanwhile, plants exposed to HS events during early (vegetative) stages (primed) can acquire basal thermotolerance and become more resistant to future HS exposure during the reproductive phases than their non-primed counterparts [197]. In addition, heat priming during early R-stages enhanced thermotolerance to post-anthesis HS in winter wheat through invigoration of photosynthesis and final grain yield [198]. Besides, heat priming imprints short-term HS memory and imparts epigenetic-modulated transgenerational memory in plants [21,38,199].

**Figure 3:**
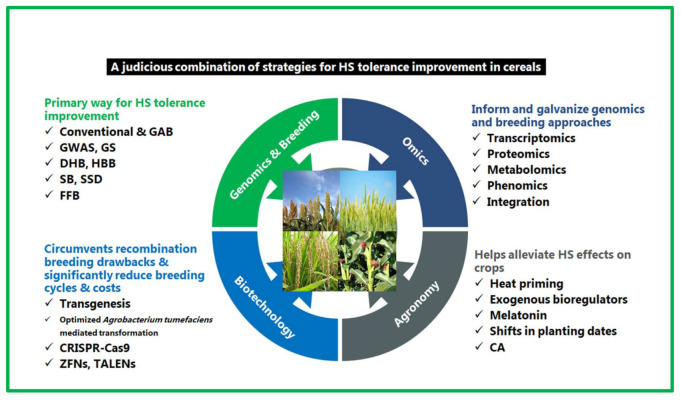
A multi-pronged approach for reproductive-stage HS tolerance improvement in cereals. Driven by advances in genome sequencing and assembly technologies, combined conventional and modern plant breeding approaches remain the primary way through which complex quantitative traits, such as HS, can be improved. Meanwhile, the advent of omics technologies has helped to inform and galvanize genomics and plant breeding programs. Biotechnological interventions such as transgenesis considerably circumvent some of the shortcomings of the recombination breeding methods, while genome-editing techniques such as CRISPR-Cas9 significantly shorten breeding cycles andreduceg breeding costs. These endeavors are supported by other disciplines such as machine learning, bioinformatics and data analytics, robotics, crop modelling and decision support systems. In addition, agronomic options remain relevant in alleviating H+D stress effects on crops in the field. **Abbreviations**: HS, heat stress; GAB, genomics assisted breeding; GWAS, genome-wide association studies; GS, genomic selection; SB. Speed breeding; DHB, doubled-haploid breeding; HBB, haplotype-based breeding; FFB, fast-forward breeding; SSD, single-seed descent; CRISPR-Cas9, clustered regularly interspaced palindromic repeats (CRISPR)–CRISPR-associated protein 9; ZFNs, zinc-finger nucleases; TALENs, transcription activator-like effector nucleases; CA, conservation agriculture; H+D, combined heat and drought.

Other HS management strategies include exogenous supplementation of phytohormones, osmoprotectants and bioregulators [27,30,34,195]. Especially, fine-tuning the action of BRs has the potential to enhance the expression of other growth promoters, amplify plant HS responses, and improve crops’ HS acclimation for growth and productivity sustenance [104]. Melatonin also boosts plant stress tolerance by suppressing HS-induced damages (such as photosynthesis inhibition), improving antioxidant defense capabilities, and enhancing stress-responsive gene expression [200]. Osmoprotectants (spermidine, glycine betaine, proline, etc.) reduce HS-induced oxidative damage and increase photosynthesis, thereby improving plant growth under HS [30,103]. Equally, boosting plant growth-promoting bacteria via bacterial seed treatment helps ameliorate HS by enhancing both seedling survival and plant HS tolerance at the subsequent R stage [27,201]. In summary, these several agronomic interventions may augment the inherent genotypic capabilities of different cereal crop cultivars in coping with HS conditons, and support other strategies (Figure 3).

## 6. Aspects Guiding Future Research Directions

Here, based on several shortcomings in current RSHS tolerance investigations in cereals and some knowledge gaps on the subject, we highlight the key aspects to guide future research priorities on RSHS tolerance in cereal crops.

### 6.1. Shortcomings in RSHS Tolerance Investigations in Cereals

Despite progress in revealing plant HS responses, several issues still need to be resolved. First, most researchers have often generalized the term “heat stress”, yet it comprises various experiment types, including **heat warming**, heat wave, and heat shock [31]; each of which has its own meaning (see Box 1) and experiment set-up parameters, depending on the magnitude and duration of the temperature elevation imposed [31]. Therefore, generalizing the term “heat stress” may be misleading. Whereas variability in heat research studies is inevitable, it is crucial for researchers to provide explicit descriptors of their heat experiment type. Thus, correct and consistent use of terms, corresponding experimental protocols, and integration of tools and results across these approaches are essential to better understand the mechanisms underlying plant HS responses [31]. Additionally, few studies have monitored plant **HS recovery** (see Box 1) in their experiments, yet it helps us better understand plant HS responses [39]. It is therefore critical that HS recovery be integrated into studies and crop breeding programs in order to complement recent progress in crop HS tolerance improvements [31]. This is particularly more relevant for future understanding of a warming climate whereby subsequent HS events may occur before the plant system has recovered from the previous event.

Second, a large majority of studies on plant HS response have focused on the vegetative and seedling stages [16]. Yet, in cereals, the R stage is the most sensitive and crucial if we are to deduce more meaningful insights. Findings obtained from such stages, when inferred to the R stage, may be misleading because the R stage is unique with its own characteristics. Therefore, more studies targeting the R stages are needed to provide much insight into RSHS response in cereals. Fortunately, we can now leverage omics technologies to perform comparative analyses of different crop genotypes’ responses to HS and identify key candidate genes or proteins underpinning those responses [189].

Third, the stress-combinatorial (multi-factorial) effects are understudied. As alluded to in the Introduction, HS seldom exists independently under field conditions but rather co-occurs with other abiotic and biotic stresses [14,16]. In particular, H+D stresses co-occur and have to be considered simultaneously since their combined effect is greater than when considered individually [9]. Generally, H+D stresses are controlled by multiple (and often similar) genes with complex underlying GRNs. Additionally, other abiotic or biotic stresses exert additive influence on the H+D combination, making it even more puzzling to investigate [9]. Therefore, experimental designs addressing multiple concurring stresses should be prioritized at the expense of sole stress experiments in order to obtain a detailed understanding of how plants respond to HS vis-à-vis other stress factors.

Fourth, delineating the R stage in cereals is still a challenge, yet this is essential for phenotyping and modelling HS responses. The R stage comprises different phases that are difficult to separate (Box 1). Especially, much of the commencement of the R stage is not visible until the sudden emergence of the ear or spikelet from the flag-leaf sheath, underlined by the rapid elongation of the peduncle [37]. Therefore, it is difficult to time and identifies a specific stage for targeted HS imposition and investigation. Thus, the accurate identification of crop growth phases is critical for distinguishing stage-specific HS effects and responses and ensuring HS regimes applied in studies are focused on the specific stage/s earmarked for investigation. This helps in providing a more detailed understanding of the responses of different crop growth stages to HS [12]. To address this, we suggest that sensor-based phenomics may play a huge role in helping identify and differentiate these unique stages for correct and properly targeted studies.

Fifth, most studies on HS (with the ultimate aim to understand or enhance plant performance in the field) are performed under controlled environments such as in growth chambers or greenhouses, which may not be correlated or readily applicable to field-grown environments [202]. In such situations, the aspect of how results from the controlled environments are translated to the field conditions is often overlooked [202]. As such, for the genetic dissection of HS tolerance in cereals, large-scale experiments in the actual crop field environments are necessary [82]. Here, improved crop phenotyping techniques such as sensor-based high-throughput plant phenotyping (HT3Ps) platforms will play a huge role in the physiological characterization of genotypes in the field [203]. Furthermore, experiments need to be designed to mimic real-life conditions to yield information that breeders can employ to develop crops tolerant to these scenarios [16].

Sixth, a large majority of plant HS response studies have used the ambient (air) temperature instead of the tissue temperatures. Usually, the ambient, canopy, and within canopy or tissue (leaf, ear, tassel, etc.) temperatures substantially differ [2]. As such, basing our HS response analyses on ambient temperature thresholds generated in chamber studies may not provide accurate results on the HS effect on specific plant tissues and their responses. Therefore, there is a growing need to link plant responses to tissue temperatures [31]. Furthermore, the nature of interactions between CO_2_, heat and drought stresses, and the connections between cropping intensity and HS exuded from heat transfer processes occurring in sparse canopies (where sensible heat and soil heat fluxes tend to dominate) should be dissected [2].

Moreover, most of the studies on HS tolerance in cereals have used model plant Arabidopsis, and not cereal model species. Therefore, conjecturing that the results obtained in Arabidopsis will have similar inferences in cereals cannot be more accurate unless these findings are translated from Arabidopsis to cereal crop species and verified under mimic or actual field conditions [204]. However, cereals suffer tissue culture and transformation limitations; they are recalcitrant to tissue culture, genotype-dependent, have longer regeneration time, and have low transformation efficiency [204,205]. Fortunately, these transformation drawbacks can now be overcome by deploying improved protocols, including optimized *Agrobacterium*-mediated transformation using immature embryos [205]. In addition, GETs such as the CRISPR-Cas9 system offer an unprecedented opportunity to bypass these tissue culture and transformation drawbacks in cereals [188].

### 6.2. Knowledge Gaps and Emerging Trends

Beyond these shortcomings, we also advance that a large knowledge gap about crop belowground responses to HS exists. This dearth of full understanding of belowground responses partly emanates from an undue emphasis on aboveground responses. Such biases potentially make it difficult to parse out vital composite plant HS responses since underground components, including roots and rhizosphere microbes, also play a part. Therefore, we propose that plant HS response studies also prioritize understanding of root responses, interactions and feedback mechanisms that exist among the rhizosphere, root microbes, and aboveground components responding to HS.

Additionally, there is a current renewed interest in understanding the effects of high night temperature (HNT) on cereals [206,207,208]. Asymmetric warming causing a rapid increase in nocturnal versus diurnal temperatures has been observed and is predicted to continue, posing significant challenges to cereal crop productivity [207,208]. Yet, our knowledge of the impacts of HNT, and tolerance mechanisms in cereals is still limited [207]. Therefore, this topic warrants further research. Priority areas include the establishment of unified and reproducible experimental protocols for HNT characterization in cereals; physiological and molecular characterization of genotypes for HNT tolerance; surveying mapping populations for natural variation for HNT tolerance at the R stage; using omics approaches to identify HNT tolerance genotypes in comparative studies; employing field-based HT3Ps to evaluate large mapping populations for HNT tolerance; detection of QTLs for yield stability under HNT conditions; discovery of common HNT responses among cereal crops; and establishing the combinatorial effects of HNT with other abiotic stresses (detailed in refs. [207,208]). This, eventually, helps provide an in-depth understanding of plant HS responses and guides inclusive RSHS tolerance improvement in cereals.

Furthermore, the trade-off dynamics that exist between the plant HS response and growth and reproduction needs underpin plants’ survival and productivity [209]. Spatiotemporal regulation of energy reserves is critical for optimizing the growth-defense trade-offs in plants [209]. Yet, the mechanisms governing these dynamics still remain largely unknown. Therefore, understanding the regulatory network underlying growth-control and HS-response pathways will be essential for resetting the balance between HS-response and growth in order to engineer crops exhibiting both higher yields and enhanced HS tolerance [210,211].

## 7. Conclusions

RSHS presents serious challenges to cereal crops’ performance and productivity by affecting several reproductive organs and processes. Therefore, the research on RSHS in cereals should be prioritized. Meanwhile, plant HS responses encompass diverse mechanisms instituted at various levels, from the whole plant to cellular and morphological to molecular. Deepening our understanding of the molecular mechanisms governing RSHS responses helps us to design effective strategies for HS tolerance improvement in cereal crops. These strategies include GAB, which has been boosted by advances in genome sequencing; modern plant-breeding methods such as GS and speed breeding, which is significantly shortening breeding cycles and saving breeding costs; biotechnological interventions and omics approaches that are galvanizing the GAB approaches, and GETs such as CRISPR-Cas9, which can help circumvent the transformation hurdles associated with *Agrobacterium*-mediated protocols in recalcitrant species. In addition, agronomic options help alleviate HS effects on crops. We underline that the integrated application of these strategies remains the best foot forward in developing RSHS-resilient cereal crop cultivars that can meet future food and feed needs. Overall, our current review furthers our understanding of RSHS responses and supports the rational design of HS-tolerant cereal crop cultivars. Moreover, we advance that overcoming the identified shortcomings in HS response investigations in cereals by addressing the identified knowledge gaps and pursuing the emerging hot topics in this subject area will provide further meaningful insights into plant HS responses and facilitate the efficient breeding of HS-tolerant cereal cultivars.

## Figures and Tables

**Table 1:** Plant heat stress response mechanisms.

Response	Description	Mechanisms	References
Short-term acclimation mechanisms			
Escape	It ensures plants complete their life cycle quickly during the brief period of favorable temperature conditions. It often leads to small crop yield penalties.	ToDF (EMF and LEF)	[11,66,67]
		Early maturation	[1,65]
Avoidance	Temporary, short-lived responses activated under warm ambient temperature conditions (species-dependent, e.g., ≤30 °C in Arabidopsis) via alterations in morphology and development, which may help in avoidance of future HS.	Thermomophogenesis	[12,21]
		Leaf re-orientation (hyponasty)	[1,12]
		Transpirational cooling	[1]
		Reversion of phyB from its active Pfr to inactive Pr conformation	[40,54,57]
		Temperature-dependent phase separation of ELF3 to liquid droplets	[40,61]
		PM lipid composition adjustment	[68]
		Regulation of PIF7 mRNA translation	[63]
Long-term adaptation mechanisms			
Tolerance	Maintenance of essential plant functions that contribute to the fitness of a genotype under HS. Responses instituted under mild to severe HS conditions (species dependent) to counteract damages to proteins and lipid membrane and maintain cellular homeostasis. These mechanisms enhance plant evolutionary adaptation under HS environment.	Heat sensors (CaMs, GPCRs, etc.)	[43,51]
		Signalling pathways (Ca^2+^, CDPKs, MAPK, etc.)	[3,17,42,44,45,51]
		Antioxidant enzyme activation	[4,34]
		HSFs and TFs activation	[3,4,19,41]
		Expression of HSPs, and other HS-responsive genes.	[4,17,44,53]
		Amplified thermoprotectants synthesis.	[34,44,49]
		Phytohormonal regulation	[18]
		Small RNAs, PTMs regulation	[5,20]
		Epigenetic regulation of HS memory	[5,21]

**Note:** HS, heat stress; ToDF, time-of-day flowering; EMF, early-morning flowering; LEF, late-evening flowering; phyB, phytochrome B; PIF7, phosphate interacting factor 7; ELF3, EARLY FLOWERING 3; CaMs, calmodulins; GPCRs, G protein-coupled receptors; CDPKs, Ca^2+^-dependent protein kinases; MAPK, mitogen-activated protein kinases; HSFs, heat shock factors; HSPs, heat shock proteins; TFs, transcription factors; PTMs, post-translational modifications.

**Table 2:** Examples of heat-stress-tolerant genes verified by transgenic approaches that can be targeted for RSHS tolerance improvement in cereals.

Class	Gene Name	Donor	Host	Approach	Physiological Effect	Ref/s.
HSFs	*TaHsfA6f*	Wheat	Arabidopsis	*A. tumefaciens* mediated OE	OE of TaHsfA6f gene in Arabidopsis improved tolerance to heat, drought and salt stresses; enhanced sensitivity to exogenous ABA, and increased ABA accumulation.	[84]
	*ZmHsf05*	Maize	Arabidopsis	Constitutive expression	*ZmHsf05*-OE in Arabidopsis enhanced both basal and acquired thermotolerances in transgenic plants as compared to Wt.	[171]
HSPs	*AtHSP101*	Arabidopsis	Rice	*A. tumefaciens* mediated OE	*AtHSP101* OE transgenic rice lines showed significantly better survival rates and growth performance in the recovery phase following HS.	[172]
	*OsHSP18.6*	Rice	Rice	OE	*OsHSP18.6* OE transgenic plants exhibited improved tolerance to HS and other abiotic stresses, and decreased MDA content but enhanced antioxidant enzyme activities under H+D.	[168]
CDPKs	*ZmCDPK7*	Maize	Maize	OE; Gene knock-down	ZmCDPK7-OE plants displayed higher thermotolerance, photosynthetic rates, and antioxidant enzyme activity but lower H_2_O_2_ and MDA contents than Wt plants under HS.	[76]
	*OsANN1*	Rice	Rice	OE	*OsANN1* enhanced HS tolerance in transgenic rice plants by promoting SOD and CAT activities, which modulated H_2_O_2_ flux and redox homeostasis.	[79]
	*ZmMAPK1*	Maize	Arabidopsis	OE	Transgenic Arabidopsis plants showed increased tolerance to D+H stresses by increasing proline content, decreasing MDA content, and increasing ROS scavenging.	[173]
TFs	*TabZIP60*	Wheat	Arabidopsis	*A. tumefaciens* mediated OE	Constitutive expression of the spliced form of TabZIP60 (TabZIP60s) enhanced HS tolerance in Arabidopsis, but OE of the unspliced form (TabZIP60u) did not.	[174]
	AtbZIP17	Arabidopsis	Arabidopsis	Gene knockout	bZIP17 mutant plants were sensitive to heat stress in terms of silique length and fertility compared to that of Wt Arabidopsis plants.	[100]
	OsNTL3	Rice	Rice	LOF; GOF	Loss of function mutation of OsNTL3 conferred heat sensitivity while inducible expression of the truncated form of OsNTL3 increased heat tolerance in rice seedlings.	[175]
	*ZmWRKY106*	Maize	Arabidopsis	OE	Improved the tolerance to D+H in transgenic Arabidopsis by regulating stress-related genes via ABA-signaling, and reducing ROS by enhancing SOD, POD and CAT under D+H stress.	[169]
	*SNAC3*	Rice	Rice	OE	Enhanced tolerance to HS, drought, and oxidative stresses; NAC3 significantly reduced H_2_O_2_ and MDA contents, and increased ROS homeostasis in transgenics.	[98]
	*DREB2A*	Maize	Maize	OE	*ZmDREB2A* OE transgenic plants showed enhanced thermotolerance.	[95]
Antioxidant enzymes	*MnSOD1*	Rice	Rice	*MnSOD1* K-D and OE	*MnSOD1*-knockdown plants were markedly sensitive to HS, while *MnSOD1*-OE plants had better chaperone activity and grain quality under HS compared to WT.	[176]
	*Rab7*	Rice	Rice	OE	Improved transformants’ survival rate, RWC, proline content, and antioxidant enzyme activities, but decreased MDA. Additionally, genes encoding ROS scavenging enzymes were significantly upregulated. Further, *OsRab7* OE increased rice grain yield under D+H stresses.	[74]
Ferritin	*TaFER-5B*	Wheat	Wheat and Arabidopsis	OE	Both *TaFER-5B* lacking wheat and Arabidopsis mutants showed the HS sensitivity phenotype. *TaFER-5B* OE plants showed improved tolerances to H+D, oxidative and excess iron stresses.	[177]
PhotoS. enzyme	*ZmPEPC*	Maize	Wheat	OE	Enhanced photochemical and antioxidant enzyme activities, upregulated expression of photosynthesis-related genes, delayed chlorophyll degradation, and altered contents of proline.	[167]
LMR protein	*fad7*	Arabidopsis	Rice	co-suppression of *fad*	Transgenic rice plants exhibited greater photosynthetic efficiency and chlorophyll content under HS than Wt	[72]

**Note:** HSFs, heat shock factors; HSPs, heat shock proteins; PhotoS. the enzyme, photosynthesis-related enzyme; MSR gene, male sterility related gene; LMR protein, lipid metabolism-related protein; fad, fatty acids desaturase; TFs, transcription factors; CDPKs, calcium-dependent protein kinases; OE, overexpression; K-D, knock-down; LOF, loss of function; GOF, gain of function; Wt, wild type; H+D, heat and drought combination.

## Data Availability

Not applicable.
